# *DRD* and *GRIN2B* polymorphisms and their association with the development of impulse control behaviour among Malaysian Parkinson’s disease patients

**DOI:** 10.1186/s12883-015-0316-2

**Published:** 2015-04-22

**Authors:** Shahidee Zainal Abidin, Eng Liang Tan, Soon-Choy Chan, Ameerah Jaafar, Alex Xuen Lee, Mohd Hamdi Noor Abd Hamid, Nor Azian Abdul Murad, Nur Fadlina Pakarul Razy, Shahrul Azmin, Azlina Ahmad Annuar, Shen Yang Lim, Pike-See Cheah, King-Hwa Ling, Norlinah Mohamed Ibrahim

**Affiliations:** Neurobiology and Genetics Group, Genetic and Regenerative Medicine Research Centre, Faculty of Medicine and Health Sciences, Universiti Putra Malaysia, 43400 Serdang, Selangor Malaysia; Medical Genetics Laboratory, Clinical Genetics Unit, Faculty of Medicine and Health Sciences, Universiti Putra Malaysia, 43400 Serdang, Selangor Malaysia; Department of Medicine, Universiti Kebangsaan Malaysia Medical Center, Bandar Tun Razak Cheras, 56000 Kuala Lumpur, Malaysia; Perdana University Graduate School of Medicine, Perdana University, 43400 Serdang, Selangor Malaysia; UKM Medical Molecular Biology Institute, UKM Medical Center, Jalan Ya’acob Latiff, Bandar Tun Razak, Cheras, 56000 Kuala Lumpur, Malaysia; Department of Biomedical Science, Faculty of Medicine, University of Malaya, 50603 Kuala Lumpur, Malaysia; Department of Medicine, Faculty of Medicine, University of Malaya, 50603, Kuala Lumpur, Malaysia; Department of Human Anatomy, Faculty of Medicine and Health Sciences, Universiti Putra Malaysia, 43400 Serdang, Selangor Malaysia

**Keywords:** High resolution melt analysis, DNA polymorphism, N-methyl-D-aspartate 2B, Dopamine receptor

## Abstract

**Background:**

Impulse control disorder (ICD) and behaviours (ICB) represent a group of behavioural disorders that have become increasingly recognised in Parkinson’s disease (PD) patients who previously used dopaminergic medications, particularly dopamine agonists and levodopa. It has been suggested that these medications can lead to the development of ICB through the abnormal modulation of dopaminergic transmission and signalling in the mesocorticolimbic dopaminergic system. Several studies have reported an association between polymorphisms in the dopamine receptor (*DRD*) and N-methyl-D-aspartate 2B (*GRIN2B*) genes with the development of ICB in PD (PD-ICB) patients. Thus, this study aimed to investigate the association of selected polymorphisms within the *DRD* and *GRIN2B* genes with the development of ICB among PD patients using high resolution melt (HRM) analysis.

**Method:**

We used high resolution melt (HRM) analysis to genotype 11 polymorphisms in 5 *DRD* genes [*DRD1* (rs4532, rs4867798 and rs265981), *DRD2* (*ANKK1* rs1800497, rs104894220 and rs144999500), *DRD3* (rs3732783 and rs6280), *DRD4* (rs1800443), and *DRD5* (rs144132215)] and 1 polymorphism in *GRIN2B* (rs7301328) in PD patients with (cases, n = 52) and without (controls, n = 39) ICB. Cases were obtained from two tertiary movement disorder centres [UKMMC (n = 9) and UMMC (n = 43)]. At both centres, the diagnosis of ICB was made using the QUIP questionnaire. Controls were recruited from PD patients who attended UKMMC and were found to be negative for ICB using the QUIP questionnaire.

**Results:**

The HRM analysis showed that 7 of 11 polymorphisms [*DRD1* (rs4532, rs4867798, and rs265981), *DRD2* (*ANKK1* rs1800497), *DRD3* (rs3732783 and rs6280), and *GRIN2B* (rs7301328)] exhibited a clear distinction between wild-type and variant alleles. Variants of *DRD2/ANKK1* rs1800497 (OR = 3.77; 95% CI, 1.38-10.30; *p* = 0.0044), *DRD1* rs4867798 (OR = 24.53; 95% CI, 1.68-357.28; *p* = 0.0054), *DRD1* rs4532 (OR = 21.33; 95% CI, 1.97-230.64; *p* = 0.0024), and *GRIN2B* rs7301328 (OR = 25.07; 95% CI, 1.30-483.41; *p* = 0.0097) were found to be associated with an increased risk of developing ICB among PD patients.

**Conclusion:**

Our findings suggest that polymorphisms in dopamine [*DRD1* (rs4532 and rs4867798) and *DRD2/ANKK1* rs1800497] and glutamate (*GRIN2B* rs7301328) receptor genes confer increased risk of ICB development among PD patients.

**Electronic supplementary material:**

The online version of this article (doi:10.1186/s12883-015-0316-2) contains supplementary material, which is available to authorized users.

## Background

Impulse control behaviours/disorders (ICB/ICD) are defined as complex behavioural disorders characterized by the failure to resist the temptation to perform an act that is harmful to the individual or to others [1]. ICB/ICD has been increasingly recognized in PD patients who develop behaviour disorders, such as pathological gambling (5%), hypersexuality (3.6%), compulsive shopping (5.7%), and compulsive eating (4.3%). More than a quarter of PD patients with ICB (PD-ICB) develop two or more behavioural addictions [2]. Patients can also develop other sub-syndromic ICB or ICD, such as compulsive medication use, punding, or hobbyism [3,4]. The primary risk factor for the development of PD-ICB is the use of dopaminergic medications. While some studies have shown that the development of ICB might be dose-dependent, others have suggested that genetic predisposition coupled with inherent ‘subconscious’ personality traits are the root cause of this problem [3,5].

A possible neurobiological explanation for the association of PD with the development of ICB centres on the dopaminergic system, which is involved in reward mechanisms, impulsiveness, and decision-making processes. Dopamine receptors (*DRD*) play an important role in activating or inhibiting downstream signalling in the dopaminergic pathway. DRD1 and DRD2 are expressed abundantly in the ventral striatum [6] and might mediate the motor effects of dopamine replacement therapies. DRD3 is expressed in the limbic area of the brain, and has been associated with both behavioural addictions [7] and substance use disorders [5]. Both DRD4 and DRD5 have been linked to attention deficit hyperactivity disorder (ADHD) [8].

Polymorphisms in *DRD1*, especially rs4532, have been studied widely to detect genetic associations with neuropsychiatric disorders [9]. The *DRD1* rs4532 polymorphism has been associated with several mental illnesses, including nicotine addiction [10], bipolar disorder [11], and ADHD [12]. Additionally, the T➔C nucleotide substitution located 800 bp upstream of exon 1 (rs265981) in *DRD1* has been reported to be associated with ADHD [12-14]. Genetic polymorphisms in *DRD3* rs6280 have been reported to be associated with the development of ICB in Korean PD patients [15]. The *DRD2/ANKK1* rs1800497 polymorphism is located close to the ankryin repeat and kinase domain containing-1 (*ANKK1*) gene. The *DRD2/ANKK1* rs1800497 variant causes a glutamic acid to lysine substitution in serine/threonine kinase that might affect substrate binding to the D2 receptor [16]. Moreover, the *DRD3* rs6280 variant causes a glycine to serine substitution at codon 9 [17], which results in low binding affinity to dopamine [18]. Although *DRD4* has not been linked to the development of PD-ICB, this receptor can also be activated by dopamine and has been linked to neurological and psychiatric conditions, such as schizophrenia, bipolar disorder, and addictive behaviour [19]. Additional variants that lead to non-synonymous amino acid substitutions in DRD1–5, such as *DRD1* rs4867798, *DRD2* rs104894220 and rs144999500, *DRD3* rs3732783, *DRD4* rs1800443, and *DRD5* rs144132215, have not been studied among ICB subjects and, therefore, were included in this study.

In addition to DRDs, N-methyl-D-aspartate (NMDA) receptors are ionotropic glutamate receptors that are involved in glutamate-mediated neurotransmission in the brain [20]. The NMDA receptor plays a role in PD development because the altered expression of both NMDA receptor subunits and factors that facilitate NMDA receptor activation by 1-methyl-4-phenyl-1,2,3,6-tetrahydropyridine (MPTP) can modulate glutamate release, which leads to the death of nigrostriatal dopamine neurons [21,22]. The NMDA receptor consists of NR1 (*GRIN1*) and NR2 (*GRIN2*) subunits. For the NR2 subunit, there are four subtypes—*GRIN2A*, *GRIN2B*, *GRIN2C*, and *GRIN2D* [23]. GRIN2B acts as the agonist binding site for glutamate and the predominant excitatory neurotransmitter receptor in the mammalian brain [24]. Variants in *GRIN2B* (c.366C > G, c.2664C > T and c.-200 T > C) are commonly found in Asian populations [25,26]. Other polymorphisms within *GRIN2B* in the Ashkenazi Jewish population has been reported to be associated with bipolar I disorder, which indicates the involvement of the glutamate signalling pathway [27]. Moreover, the *GRIN2B* rs7301328 variant has been reported to be associated with the development of ICD in Korean PD patients [15].

Thus, in this study we investigated the associations of selected polymorphisms in *DRDs* and *GRIN2B* with the development of ICB among PD patients using high resolution melt (HRM) analysis. We selected 11 polymorphisms within *DRD1* (rs4532, rs4867798, and rs265981), *DRD2* (*ANKK1* rs1800497, rs104894220, and rs144999500), *DRD3* (rs3732783 and rs6280), *DRD4* (rs1800443), *DRD5* (rs144132215), and *GRIN2B* (rs7301328) for screening. The association of these polymorphisms with ICB development among a cohort of Malaysian PD subjects was then evaluated.

## Methods

### Subjects and clinical measures

This study involved samples from 91 PD patients obtained from two larger clinical studies on ICB among PD patients, in two tertiary centres in Kuala Lumpur [Universiti Kebangsaan Malaysia Medical Centre (UKMMC) and Universiti Malaya Medical Centre (UMMC)]. In both centres, patients with idiopathic PD (H&Y Stages I-IV), diagnosed by two movement disorder neurologists (NMI and SYL), and on dopaminergic medications were screened for ICB using the QUIP questionnaire. The diagnosis of ICB was based on previously established criteria published elsewhere [28]. The QUIP questionnaire is a validated questionnaire for the detection of ICB in PD. It consists of 3 sections: Section 1 assesses ICDs (sexual, gambling, eating, and buying behaviour), Section 2 assesses other compulsive behaviours, such as compulsive buying, hypersexuality, punding, hobbyism, and walkabout, and Section 3 assesses compulsive medication use [28]. Cases and controls were determined based on QUIP positivity. Fifty-two cases (QUIP positive or PD-ICB) and 39 controls (QUIP negative) were selected randomly for this study. This study was conducted in accordance with the Declaration of Helsinki and was approved by Institutional Ethics Committee from both centres (UKM-DLP-2011-048 for UKMMC and MEC No: 745.81 for UMMC). Written informed consent for participation in the study was obtained from all recruited participants.

### DNA samples

Genomic DNA from blood samples was extracted from buffy coat leukocytes using a QiaAmp DNA Blood Mini Kit (Qiagen, Limburg, The Netherlands) according to the manufacturer’s protocol. The concentration and purity (1.7–1.9) of DNA samples were measured using NanoVue™ Plus (GE Healthcare, Berkshire, UK), and the integrity of DNA was analysed using 0.8% agarose gel electrophoresis.

### Assay design and polymerase chain reaction optimization

Ten primer sets were designed using Primer3Plus to detect 11 polymorphic loci in *DRD1* (rs4532 and rs4867798), *DRD2* (*ANKK1* rs1800497, rs104894220, and rs144999500), *DRD3* (rs3732783 and rs6280), *DRD4* rs1800443, *DRD5* rs144132215 and *GRIN2B* rs7301328, which were mined from the National Center for Biotechnology Information (NCBI) database. The primer set for DRD1 rs265981 was synthesized based on a previous study [29]. Secondary structure was predicted using DINAMelts software, which also was used to calculate the melting temperature [30]. All primers were optimized using gradient polymerase chain reaction (PCR) and real-time PCR prior to HRM analysis. Primer sequences and properties are detailed in Table 1.

**Table 1:** **Loci of selected** ***DRD*** **and** ***GRIN2B*** **SNPs and the corresponding primer pairs used for high resolution melt analysis**

**SNP**	**Chromosome**	**Location**	**Allele changes**	**Amino acid changes**	**Forward primer (5′➔3′)**	**Reverse primer (5′➔3′)**	**Amplicon size (bp)**
***DRD1*** **rs4532**	chr 5 (q35.2)	19681423	C > T	Nil	GAACAGAGAAGTCCCTCTCCAC	CTGGAAATCTGACTGACCCCTA	147
***DRD1*** **rs4867798**	chr 5 (q35.2)	19679572	T > C	Nil	GGGCTCTTCTTAAGTTGGCTTT	GGACACAGATAAATGCAAGGTG	189
***DRD1*** **rs265981**	chr 5 (q35.2)	174870940	T > C	Nil	GCTCTCTCCCAAGGAAGCTC	GTGCGTTTGGGGAAAGGATC	141
***DRD2/ANKK1*** **rs1800497**	chr 11 (q23.2)	16833244	C > T	Glu > Lys	CTCTAGGAAGGACATGATGCCC	GCAACACAGCCATCCTCAAAG	128
***DRD2*** **rs104894220**	chr 11 (q23.2)	16850073	G > A	Val > Ile	CATGCCCATGCTGTACAATACG	GTACCTGCGTTATTGAGTCCGA	126
***DRD2*** **rs144999500**	chr 11 (q23.2)	16845792	G > A	Pro > Leu	GAGCATCTGAGTGGCTTTCTTCTC	GAGAAGAATGGGCATGCCAAAG	150
***DRD3*** **rs3732783**	chr 3 (q13.31)	20385935	T > C	Ala > Ala	AGTAGGAGAGGGCATAGTAGGC	CTGGGCTATGGCATCTCTGAG	116
***DRD3*** **rs6280**	chr 3 (q13.31)	20385961	C > T	Gly > Ser	AGTAGGAGAGGGCATAGTAGGC	CTGGGCTATGGCATCTCTGAG	116
***DRD4*** **rs1800443**	chr 11 (p15.5)	579830	T > G	Val > Gly	TACTGTGCGGCCTCAACGAC	GGGTAGGAAGAAGGAGCACAC	104
***DRD5*** **rs144132215**	chr 4 (p16.1)	966018	G > T	Gly > Trp	GTCCATCCTCATCTCCTTCATTCC	CTGGAGTCACAGTTCTCTGCAT	159
***GRIN2B*** **rs7301328**	chr 12 (p13.1)	6778901	G > C	Pro > Pro	CTCCCTGCAGCCCCTTTTTA	CGCCCAGATCCTCGATTTCA	109

### High resolution melting-Polymerase chain reaction

The HRM analysis was carried out in 10 μl reaction volumes containing 30 ng genomic DNA, 0.7 μM primers, and 1× Type-It HRM-PCR master mix (HotStarTaq Plus DNA Polymerase, EvaGreen Dye, and an optimized concentration of Q-solution, dNTPs, and MgCl_2_; Qiagen). The primer pairs used for HRM analysis are summarised in Table 1. Reactions were run on a 5-plex HRM real-time PCR machine (Rotor-Gene™ 6000, Qiagen) consisting of 95°C for 5 min for initial denaturation, 45 cycles of 95°C for 10 sec and 60°C for 30 sec followed by a high resolution melting phase (65-90°C for 2 sec). Data were analysed using Rotor-Gene 6000 Series Software 1.7. To ensure accuracy, raw data were selected according to the following criteria.

i. The C_t_ value and amplification rate of the samples based on comparative quantitative analysis must be less than 30 cycles and more than 1.4 cycles, respectively.
ii. Samples must show a single peak in a derivative melt-curve analysis ranging from 75-85°C.
iii. The confidence value for each genotype prediction must be greater than 90% when compared to the genotype based on reference samples and confirmed by DNA sequencing analysis.

### Sequencing

A total of 17% of samples were selected for DNA sequencing analysis to confirm the HRM genotyping results. Samples selected from each variant cluster were purified using a Fragment DNA purification kit (Intron Biotechnology, South Korea) followed by Sanger sequencing analysis to confirm the nucleotide polymorphisms. Sequences were analysed and alignments were performed using DNA Baser v3.5.4 software [31]. A Phred score of 20 or greater was used to indicate high quality sequencing results.

### Data and statistical analysis

SNPStats [32] was used to assess genotypic frequencies, Hardy–Weinberg equilibrium (HWE) and linkage disequilibrium (LD) among PD patients with or without ICB. The HWE test was performed to examine the genotypic distributions of polymorphisms in PD-ICB patients. The HWE deviation of allele and genotype frequencies was assessed by Fisher’s exact analysis. The logistic regression was used to estimate the odds ratio (OR) and 95% confidence interval (CI) for associations between each locus and the presence of PD-ICB. The analysis was used to estimate the regression coefficient and the association in the log odds of the subject with a polymorphism. The association with disease is modelled depending on the response variable. The variable response was defined as a binary response (categorical variable) and logistic regression was used to assess the proportion of variation in the response between the polymorphisms and other factors such as gender, age, ethnicity, dosage of medication and duration of PD. The association was based on co-dominant, dominant, recessive, overdominant, and log-additive models between each polymorphism in PD-ICB patients. The right model was selected based on the significant *p* value and the lowest Bayesian Information Criterion (BIC) score. Pair-wise LD statistics (D’ and r^2^) were used to determine the robustness of LD between polymorphisms. In all statistical analyses, comparisons were considered to be significant when *p* < 0.0167 after Bonferroni correction for multiple comparisons [33].

## Results

### Demographic analysis of subjects

A total of 91 PD patients with (QUIP positive or cases; n = 52) and without ICB (QUIP negative or controls; n = 39) were recruited from a total of 280 PD patients from two medical centres and were analysed. Comparison between the cases and controls in terms of age, sex and racial distribution is presented in Table 2. The mean disease duration for the cases and controls were 8.2 ± 0.7 and 5.8 ± 0.7 years, respectively. Most PD-ICB patients received a combination therapy consisting of levodopa and dopamine agonist (54%) followed by levodopa monotherapy (29%). In the control group, 39% of patients received levodopa alone whereas 36% of patient received a combination of levodopa and dopamine agonist. The other control (15%) and PD-ICB (13%) patients received dopamine agonist alone. The mean daily levadopa dose and dopamine agonist dose were higher in the cases than the controls (Table 2).

**Table 2:** **The demographic and clinical characteristics of ICB in PD patients**

**Variables**	**Control (n = 39)**	**Case (n = 52)**
**Age**		
**Mean ± SE**	63.46 ± 1.344	62.42 ± 1.087
**Median**	63.00	63.00
**Range**	42 - 85	42 - 77
**Duration of PD**		
**Mean ± SE**	5.77 ± 0.745	8.17 ± 0.725
**Median**	4.00	8.00
**Range**	1.0 - 20.0	0.0 - 23.0
**Gender, n (%)**		
**Male**	27 (69)	38 (73)
**Female**	12 (31)	14 (27)
**Ethnicity, n (%)**		
**Malay**	13 (33)	8 (15)
**Chinese**	25 (64)	34 (66)
**Indian**	1 (3)	8 (15)
**Others**	0 (0)	2 (4)
**Drug Medication, n (%)**		
**No Medication**	4 (10)	2 (4)
**Levadopa only**	15 (39)	15 (29)
**Dopamine agonist only**	6 (15)	7 (13)
**Levadopa + Dopamine agonist**	14 (36)	28 (54)
**Dosage of Levadopa**		
**Mean ± SE**	172.79 **±** 26.89	345.98 **±** 41.53
**Median**	150.00	150.00
**Range**	0.00 - 675.00	0.00 - 675.00
**Dosage of DA**		
**Mean ± SE**	1.05 ± 0.19	82.79 ± 12.16
**Median**	1.00	80.00
**Range**	0.00 - 5.00	0.00 - 360.00
**Diagnosis, n (%)**		
**One repetitive behaviour**	0 (0)	30 (57)
**>1 repetitive behaviour**	1 (3)	15 (29)
**Compulsive medication**	1 (3)	2 (4)
**>1 repetitive behaviour + Compulsive medication**	0 (0)	5 (10)
**No repetitive behaviour + Compulsive medication**	37 (94)	0 (0)

### High resolution melt analyses

**Figure 1:**
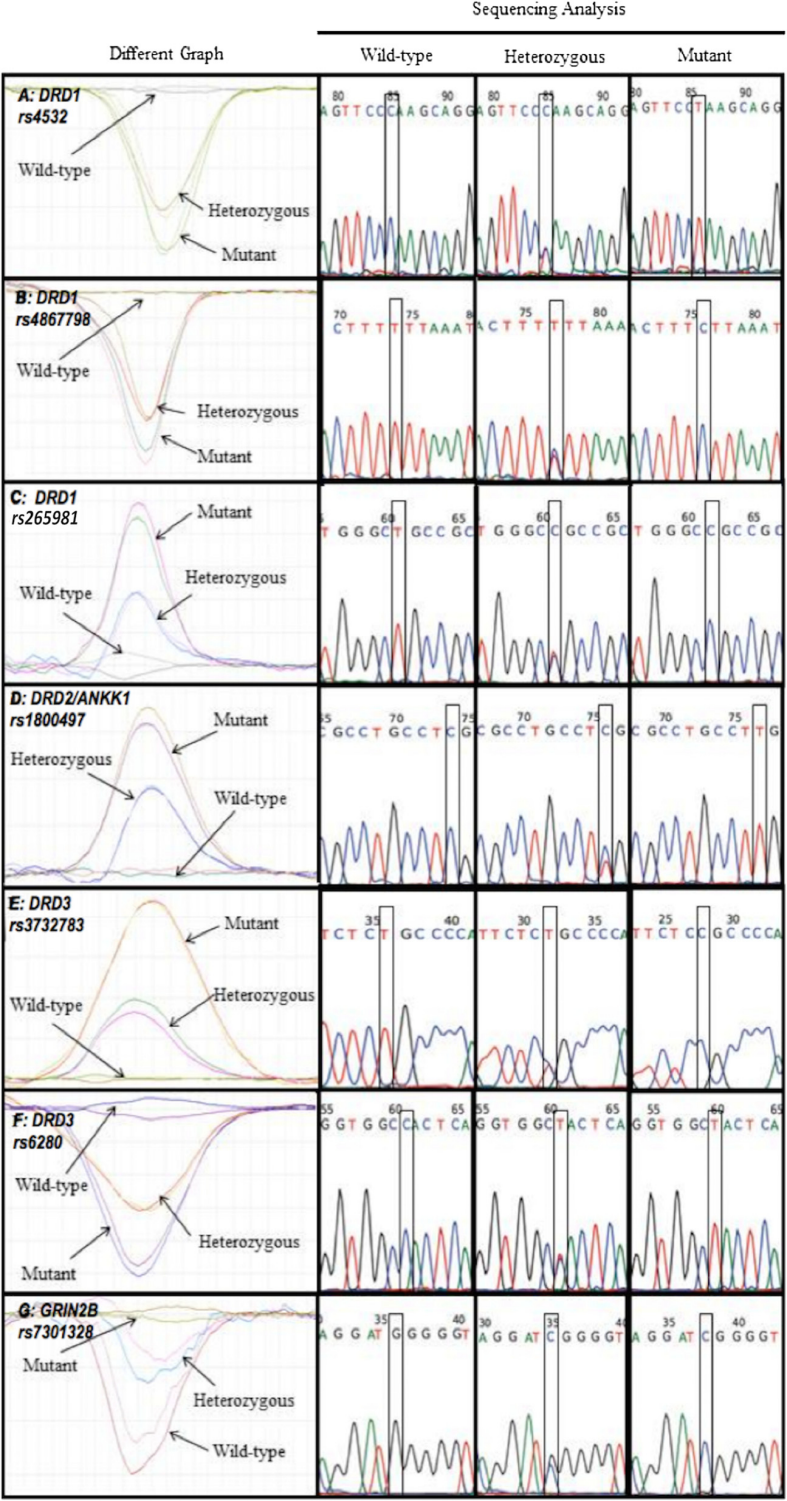
The HRM profiles and sequence analyses of **(a)** DRD1 rs4532, **(b)** DRD1 rs4867798, **(c)** DRD1 rs265981, **(d)** DRD2/ANKK1 rs1800497, **(e)** DRD3 rs3732783, **(f)** DRD3 rs6280, and **(g)** GRIN2B rs7301328, which are presented along with their respective sequencing results.

In this study, 11 polymorphisms were analysed, but only 7 polymorphisms showed allelic variation [*DRD1* (rs4532, rs4867798, rs265981), *DRD2/ANKK1* rs1800497, *DRD3* (rs3732783 and rs6280), and *GRIN2B* rs7301328] (Figure 1). The other 4 polymorphisms [*DRD2* (rs104894220 and rs144999500), *DRD4* rs1800443, and *DRD5* rs144132215] screened were found to be monomorphic (Additional file 1: Figure S1). We have successfully established 11 HRM assays for the detection of polymorphisms in *DRD1*-*5* and *GRIN2B*. The HRM assays were very efficient, sensitive, and specific in identifying nucleotide transitions (C > T or T > C) and transversions (G > C) in our samples. Approximately 17% of the total numbers of HRM reactions were sequenced and the results were 100% in agreement with the HRM profiles. The differential melting curves for the 7 corresponding polymorphisms (C > T, T > C, or G > C) clearly distinguished the wild-type from the variant genotypes. The amplicons derived from the *DRD1* rs4532, *DRD2*/*ANKK1* rs1800497, and *DRD3* rs6280 mutant alleles showed a single nucleotide change from C to T. Polymorphisms in *DRD1* rs4867798, *DRD1* rs265981, and *DRD3* rs3732783 showed a single nucleotide change from T to C. Analysis of the amplicons derived from *GRIN2B* rs7301328 showed a single nucleotide change from G to C. All analysed SNPs were deposited in dbSNP build B145 (http://www.ncbi.nlm.nih.gov/SNP/) with the following accession numbers; ss1713988434 (rs265981), ss1713988435 (rs4532), ss1713988436 (rs4867798), ss1713988437 (rs104894220), ss1713988438 (rs144999500), ss1713988439 (rs1800497), ss1713988440 (rs3732783), ss1713988441 (rs6280), ss1713988442 (rs1800443), ss1713988443 (rs144132215) and ss1713988444 (rs7301328).

### Genotype distribution in Parkinson’s disease–impulse control disorder patients

No significant deviation was observed in the distribution of genotype and allele frequencies for all polymorphisms (except for *DRD3* rs3732783) based on HWE analysis between the case and control groups (Table 3). The frequency of the C allele for *DRD3* rs3732783 was significantly higher (*p* < 0.01) in the PD population. Moreover, pair-wise LD measurements between polymorphisms in *DRD1* based on the Dʹ statistic showed no difference in LD among the polymorphisms (rs4532, rs4867798, and rs265981). By contrast, as expected, *DRD3* showed strong LD (D′ > 0.99) between polymorphisms (rs3732783 and rs6280) that could be observed using multiple-SNP analysis (Table 4). In multiple haplotype analysis of *DRD1* and *DRD3*, we detected low frequencies for all alleles (Additional file 2: Table S1).

**Table 3:** **The distribution of polymorphisms in the case and control groups**

**SNP**		**Allele frequency**		**Genotype frequency**			***p*** **-value**
				**Wild type**	**Heterozygote**	**Mutant**	
***DRD1*** **rs4532**	Control	C (0.06)	T (0.94)	CC (0.02)	TC (0.07)	TT (0.90)	0.26
	Case	C (0.24)	T (0.76)	CC (0.06)	TC (0.37)	TT (0.57)	
***DRD1*** **rs4867798**	Control	T (0.68)	C (0.32)	TT (0.42)	TC (0.52)	CC (0.06)	0.35
	Case	T (0.54)	C (0.46)	TT (0.36)	TC (0.36)	CC (0.28)	
***DRD1*** **rs265981**	Control	T (0.18)	C (0.82)	TT (0.03)	TC (0.32)	CC (0.66)	0.51
	Case	T (0.21)	C (0.79)	TT (0.02)	TC (0.36)	CC (0.66)	
***DRD2/ANKK1*** **rs1800497**	Control	C (0.48)	T (0.52)	TT (0.30)	TC (0.44)	CC (0.26)	0.27
	Case	C (0.71)	T (0.29)	TT (0.09)	TC (0.40)	CC (0.51)	
***DRD3*** **rs3732783**	Control	T (1.00)	C (0.00)	TT (1.00)	TC (0.00)	CC (0.00)	0.0014^**^
	Case	T (0.95)	C (0.05)	TT (0.94)	TC (0.02)	CC (0.04)	
***DRD3*** **rs6280**	Control	C (0.24)	T (0.76)	CC (0.05)	TC (0.50)	TT (0.57)	0.61
	Case	C (0.33)	T (0.76)	CC (0.08)	TC (0.38)	TT (0.42)	
***GRIN2B*** **rs7301328**	Control	G (0.50)	C (0.50)	GG (0.57)	CG (0.38)	CC (0.05)	0.47
	Case	G (0.28)	C (0.72)	GG (0.42)	CG (0.50)	CC (0.08)	

Data were analysed using Fisher’s exact test. ^**^ **,** *p* < 0.01, which indicates a statistically significant difference.

**Table 4:** **Pairwise linkage disequilibrium among** ***DRD1*** **and** ***DRD3*** **variants by D’ statistic**

**DRD1**	**rs4532**	**rs4867798**	**rs265981**
**rs4532**	-	0.250	0.789
**rs4867798**	-	-	0.011
**rs265981**	-	-	-
**DRD3**	**rs3732783**	**rs6280**	
**rs3732783**	-	0.996	
**rs6280**	-	-	

### Association of *DRDs* and *GRIN2B* polymorphisms with Parkinson disease–Impulse control disorder

We evaluated the associations between polymorphisms in *DRD* and *GRIN2B* with ICB risk among Malaysian PD patients (Table 5). Among the 7 polymorphisms examined, only 4 were associated with the development of ICB among PD patients. Logistic regression analysis (adjusted for gender, age, ethnicity, dosage of medication and duration of PD) showed that there was a significantly higher risk of ICB that was associated with the *DRD1* rs4867798 C allele (OR = 24.53; 95% CI, 1.68-357.28; *p* = 0.0054) and the *GRIN2B* rs7301328 C allele (OR = 25.07; 95% CI, 1.30-483.41; *p* = 0.0097). Similarly, the *DRD1* rs4532 T allele (OR = 21.33; 95% CI, 1.97-230.64; *p* = 0.0024) and T allele in *DRD2/ANKK1* rs1800497 (OR = 3.77; 95% CI, 1.38–10.30; *p* = 0.0044) were significantly associated with a higher risk of developing ICB in PD patients. The *DRD1* rs4867798 and *GRIN2B* rs7301328 polymorphisms showed a recessive mode of inheritance, suggesting that the dominant allele has an advantage over the recessive allele. Notably, *DRD1* rs4532 showed an overdominant mode of inheritance in which heterozygous patients (TC) have an increased risk of ICB compared to homozygous patients (CC or TT). By contrast, the T allele in *DRD2*/*ANKK1* rs1800497 was shown to have an additive genetic effect, whereby the risk of a patient with 2 copies of the T allele doubles compared to a heterozygous patient. No association was detected between the other polymorphisms and ICB in PD patients.

**Table 5:** **Association between PD-ICB and polymorphisms in genotypes among cases and controls**

**Model**	**Genotype**	**Control (%)**	**Case (%)**	**OR (95% CI)**
***DRD1*** **rs4532 (C > T)**				
**Overdominant**	CC-TT	35 (94.6)	32 (61.5)	1.00
	TC	2 (5.4)	20 (38.5)	21.33 (1.97-230.64)^**^
***DRD1*** **rs4867798 (T > C)**				
**Recessive**	TT-TC	29 (96.7)	32 (71.1)	1.00
	CC	1 (3.3)	13 (28.9)	24.53 (1.68-357.28)^**^
***DRD2/ANKK1*** **rs1800497 (C > T)**				
**Log-additive**	T-	-	-	3.77 (1.38-10.30) ^**^
***GRIN2B*** **rs7301328 (G > C)**				
**Recessive**	GG-CG	24 (82.8)	20 (47.6)	1.00
	CC	5 (17.2)	22 (52.4)	25.07 (1.30-483.41) ^**^

^**^ **,** *p* < 0.01, which indicates a statistically significant difference.

## Discussion

This study showed that the rs4532 and rs4867798 variants in *DRD1* were associated with ICB in PD patients. *DRD1* encodes one of the major receptors in the brain that mediate the actions of the neurotransmitter dopamine in various psychomotor functions [10]; data suggest that polymorphisms in the promoter region of *DRD1* may play a role in the neurobiology of ICB [29]. Previous studies have shown that the rs4532 polymorphism in the 5′-UTR is significantly associated with compulsive addictive behaviour [11,34,35]. These studies have shown that the T allele of rs4532 imparts a higher risk of developing compulsive addictive behaviour in healthy subjects. Similarly, our study showed that this allele was significantly associated with an increased risk of developing ICB in PD patients. To date, there has been no report that the rs4867798 polymorphism in the 3′-UTR of *DRD1* was associated with ICB. However, we found that the C allele of rs4867798 was significantly associated with a greater risk of developing ICB in PD patients. In our haplotype-based analysis, we detected no LD between rs4532 and rs4867798 in *DRD1*. Therefore, the rs4532 and rs4867798 variants were independently associated with the development of ICB among PD patients.

Both the rs4532 and rs4867798 polymorphisms are located outside of the coding region of *DRD1*. However, to date, no polymorphism has been found to alter the amino acid sequence in the *DRD1* coding region [36]. Thus, these polymorphisms located in the 3′- and 5′-UTRs of *DRD1* are likely to affect mRNA stability, and to subsequently affect *DRD1* expression [10]. It is possible that these two polymorphisms, rs4532 and rs4867798, interfere with mRNA stability, which could affect the binding site of microRNA (miRNA) or change the secondary structure of mRNA. However, further experiments will be needed to test these possibilities.

Among all polymorphisms in *DRD2/ANKK1*, rs1800497 has been the most frequently implicated in addiction disorders [37]. This polymorphism was previously reported to be associated with cocaine addiction and pathological gambling in the general population [38,39]. In our study, the *DRD2/ANKK1* rs1800497 variant was shown to be associated with PD-ICB, which was consistent with a previous study of PD-ICD subjects [40]. By contrast, Lee et al. [15] and Vallelunga et al. [41] showed that there were no associations of *DRD2/ANKK1* rs1800497 variants with ICB/ICD among PD subjects. DRD2 is involved in the mesocorticolimbic pathway, which is mainly distributed in the striatum [16] and also affects motor control [15]. The rs1800497 variant in *DRD2/ANKK1* has been associated with decreased receptor density in the striatum [42]. In agreement with these speculative points [16,17,41], the rs1800497 variant in *DRD2/ANKK1* changes the glutamic acid to lysine (from an amino acid group with a negatively charged side chain to positively charged residue) that might result in a significant protein structure modification that leads to reduced expression of the receptor and the development of neuropsychiatric disorders among PD patients. Therefore, molecular studies of the effect of *DRD2/ANKK1* rs1800497 variants should be explored.

In *GRIN2B*, the rs7301328 variant was previously found to be associated with PD under a dominant model [40]. In our study, we found that the same polymorphism exhibited an increased risk of developing ICB among PD subjects and was in accord with a study conducted by Lee et al. [15]. The rs7301328 variant causes a synonymous single nucleotide substitution. It alters the DNA sequence, but does not change the encoded amino acid sequence. This polymorphism has been found to be associated with alcohol dependence [43] and schizophrenia [26,44]. The association between the NMDA receptor subunits based on polymorphisms in *GRIN1* and *GRIN2B* and PD-ICB has not been thoroughly explored. Thus, screening for polymorphisms in the *GRIN1* subunit and subtypes of *GRIN2* genes could provide important insights into the understanding of gene-to-gene interactions that influence ICB among PD subjects.

## Conclusion

In summary, we have shown that variants in *DRD1* rs4867798, *DRD1* rs4532, *DRD2/ANKK1* rs1800497 and *GRIN2B* rs7301328 are associated with an increased ICB risk among PD patients. Future studies of gene-to-gene interactions and also identifying the miRNA binding site domains could yield an improved understanding of how synonymous polymorphisms can lead to ICB development. Furthermore, the combinatorial effects of individual polymorphisms in genes that participate in the dopaminergic and glutamategic pathways should be examined. Additional assessment by psychiatrists will relate the association of these ICB susceptible polymorphisms to ICD.

## Availability of supporting data

Two additional files were provided as supporting data. Additional file 1: Figure S1 contains a supplementary figure that depicts high resolution melting normalised curves and DNA sequencing results for selected *DRD2*, *DRD4* and *DRD5* SNPs. Additional file 2: Table S1 contains a supplementary table for haplotype analysis of selected *DRD1* and *DRD3* SNPs.

## Additional files

Additional file 1: Figure S1. A High Resolution Melting normalised curve for (A) *DRD2* rs104894220, (B) *DRD2* rs144999500, (C) *DRD4* rs1800443, and (D) *DRD5* rs144132215, which are presented along with their respective sequencing results. Additional file 2: Table S1. Haplotype analysis of selected *DRD1* and *DRD3* SNPs.
