# Supplementary material for: DRD and GRIN2B polymorphisms and their association with the development of impulse control behaviour among Malaysian Parkinson’s disease patients
Source: BMC Neurol. 2015 Apr 22;15:59. doi: 10.1186/s12883-015-0316-2 (PMC4417293; doi:10.1186/s12883-015-0316-2)

**Additional file 1: Figure S1** A High Resolution Melting normalised curve for (A) *DRD2* rs104894220, (B) *DRD2* rs144999500, (C) *DRD4* rs1800443, and (D) *DRD5* rs144132215, which are presented along with their respective sequencing results.


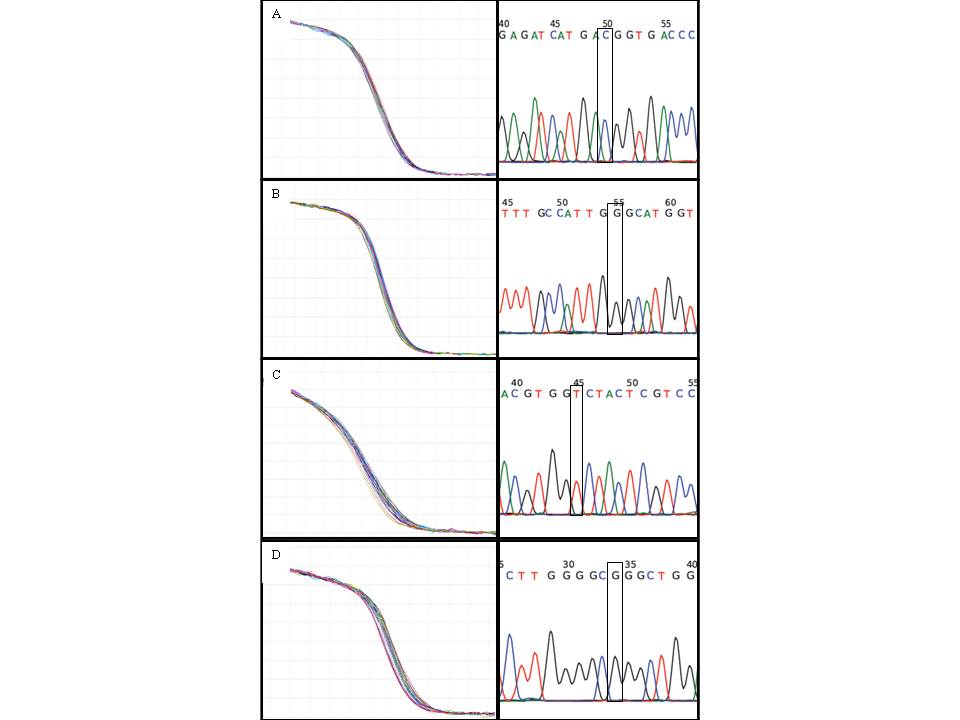

Supplement: Additional file 1: Figure S1. — A High Resolution Melting normalised curve for (A) DRD2 rs104894220, (B) DRD2 rs144999500, (C) DRD4 rs1800443, and (D) DRD5 rs144132215, which are presented along with their respective sequencing results. [file 12883_2015_316_MOESM1_ESM.doc]
