# Supplementary material for: DRD and GRIN2B polymorphisms and their association with the development of impulse control behaviour among Malaysian Parkinson’s disease patients
Source: BMC Neurol. 2015 Apr 22;15:59. doi: 10.1186/s12883-015-0316-2 (PMC4417293; doi:10.1186/s12883-015-0316-2)
Supplement: Additional file 2: Table S1. — Haplotype analysis of selected DRD1 and DRD3 SNPs. [file 12883_2015_316_MOESM2_ESM.doc]

**Additional file 2: Table S1:** Haplotype analysis of selected *DRD1* and *DRD3* SNPs

DRD1

| **Haplotype frequencies estimation (n = 97)** | | | | | | |
| --- | --- | --- | --- | --- | --- | --- |
| **rs4532** | **rs4867798** | **rs265981** | **Total** | **Control** | **Case** | **Cumulative frequency** |
| T | T | C | 0.448 | 0.544 | 0.362 | 0.448 |
| T | C | C | 0.324 | 0.283 | 0.367 | 0.772 |
| C | T | T | 0.087 | 0.041 | 0.127 | 0.859 |
| C | C | T | 0.051 | 0.027 | 0.063 | 0.910 |
| T | T | T | 0.033 | 0.105 | 0.000 | 0.943 |
| C | T | C | 0.029 | NA | 0.052 | 0.973 |
| T | C | T | 0.028 | 0.000 | 0.030 | 1.000 |

***DRD3***

| **Haplotype frequencies estimation (n = 97)** | | | | | |
| --- | --- | --- | --- | --- | --- |
| **rs3732783** | **rs6280** | **Total** | **Control** | **Case** | **Cumulative frequency** |
| T | T | 0.713 | 0.762 | 0.673 | 0.713 |
| T | C | 0.261 | 0.238 | 0.281 | 0.974 |
| C | C | 0.026 | NA | 0.046 | 1.000 |
| C | T | 0.000 | NA | 0.000 | 1.000 |
